# Comparison of Quality of Life in Breast Cancer Survivors with and without Persistent Depressive Symptoms: A 12-Month Follow-Up Study

**DOI:** 10.3390/ijerph20043663

**Published:** 2023-02-18

**Authors:** Fernanda Elisa Ribeiro, William Rodrigues Tebar, Gerson Ferrari, Mariana Romanholi Palma, Cristina Elena Fregonesi, Daniela Tanajura Caldeira, Gabriela Caroline Rodrigues Silva, Luiz Carlos Marques Vanderlei, Victor Spiandor Beretta, Diego Giulliano Destro Christofaro

**Affiliations:** 1 Physical Education Department, Graduate Program in Movement Sciences, School of Technology and Sciences, São Paulo State University (Unesp), São Paulo 19060-900, Brazil; 2 Center of Clinical and Epidemiological Research, University Hospital, University of Sao Paulo, São Paulo 05403-000, Brazil; 3 Faculty of Health Sciences, Universidad Autónoma de Chile, Providencia 7500912, Chile

**Keywords:** depression, exercise, breast neoplasms, quality of life, mental health

## Abstract

Although breast cancer treatments reduce mortality, their adverse effects can increase depression which impacts one’s quality of life (QoL). Physical activity (PA) seems to improve the QoL of breast cancer survivors (BCS). However, an unanswered question is the influence of PA on the QoL in BCS with depressive symptoms. Thus, we analyzed the influence of PA on the QoL in BCS with persistent depressive symptoms during 12 months of follow-up. The sample included 70 female BCS. Depression and QoL domains (i.e., functional capacity, physical limitations, body pain, general health status, vitality, social and emotional aspects, and mental health) were assessed at baseline and follow-up periods by the Hospital Anxiety and Depression Scale and SF-36, respectively. Habitual PA was assessed by Baecke’s questionnaire. Our results indicate a prevalence of 17.1% of depressive symptoms. Non-depressives BCS improved their physical limitations and general health status domains over time, but there were no observed differences in depressive BCS. BCS with persistent depressive symptoms (baseline and follow-up) showed worse QoL scores than non-depressives in all domains, regardless of confounding factors. When adjusted for PA, the difference between BCS depressives and non-depressives lost its significance in the functional capacity domain. In conclusion, habitual PA practice positively influenced the functional capacity domain of the QoL in BCS.

## 1. Introduction

Cancer is the most common cause of premature death, increasing the number of deaths before 70 years old [1]. Thus, it is considered an important barrier to extending life expectancy worldwide [2]. The global prevalence of cancer has risen over the past years and in 2020, approximately 19 million new cases of cancer were estimated around the world [3]. The prevalence of cancer globally is expected to increase by 47% in 2040 in relation to 2020 [3].

In men from the USA, the most prevalent types of cancer are prostate, melanoma of the skin, and colon and rectum, while the most prevalent types of cancer in women are breast, uterine, and thyroid [4]. In 2020, approximately two million new cases of female breast cancer were diagnosed worldwide [3]. Breast cancer is considered one of the main causes of death worldwide, mainly in transitioning countries [3,5], and the second most common type of cancer in the Brazilian female population [6,7]. However, post-breast cancer survival has increased substantially in recent years, mainly due to early diagnosis and treatment efficacy [8]. Although the treatments for breast cancer contribute substantially to reducing mortality, their adverse effects have negatively impacted quality of life (QoL) [8,9,10,11,12,13]. Thus, the QoL of breast cancer survivors has been widely studied, since this population experiences several physical and psychological confrontations from diagnosis to the post-treatment period [13,14,15,16].

As a result of the increase in the survival rate and prolonged life expectancy due to diagnosis and treatment efficacy, the relevance of QoL and mental health in breast cancer survivors is growing [17]. Fatigue and pain are the most frequently reported physical complaints after cancer treatment, and they have been associated with psychological disorders such as anxiety and depression [15,18,19]. Approximately 22% to 38% of patients diagnosed with breast cancer have depression symptoms, with 22% of them demonstrating moderate to severe depression levels [19,20]. Substantial evidence has shown that psychological disorders, mainly depression, have been associated with worse QoL in breast cancer survivors [13,15,16,18,21]. In this sense, depression symptoms may affect several factors related to the QoL of breast cancer survivors, such as their body image, functional capacity, mental well-being, and involvement in regular physical activity [13,15].

Increasing the practice of physical activity seems to improve functional capacity and decrease the chance of developing several side effects during cancer treatment by 26% [22]. Breast cancer patients who are more physically active present a better QoL, mainly in the functional capacity domain, than sedentary patients [23]. Physical activity is also considered indispensable for maintaining the health of breast cancer survivors due to the physical and metabolic benefits it provides. In addition, physical activity is considered an important strategy for improving QoL and positively affecting physical functioning, fatigue, pain, and sexual function [17], besides serving as a protective measure against disease recurrence [15,24,25].

Although the relationship between depression and QoL in breast cancer survivors is well-established [13,15,16,18,21], little is known about the influence of persistent depressive symptoms on the QoL of this population over time [26]. This influence should be highlighted since the physical and emotional consequences of breast cancer treatment can persist throughout the years, even after the disease has been treated [27]. Furthermore, physical activity is able to reduce the risk of disease recurrence, improve mental well-being, and prevent other comorbidities [28,29,30,31]. In addition, while the benefits of physical activity in the QoL of breast cancer survivors are known, it is not clear whether physical activity can influence the QoL domains of breast cancer survivors with symptoms of depression, especially when considering the repercussions of postoperative time. Most studies that have assessed QoL and mental health in breast cancer survivors are cross-sectional or short-term, which highlights the need for a prolonged follow-up investigation on this topic.

Therefore, the aim of this study was to compare the QoL domains of breast cancer survivors according to persistent depressive symptoms (over 12 months) and to analyze the influence of physical activity on the QoL domains in this population. We hypothesize that breast cancer survivors with depression symptoms would present lower QoL domains than non-depressive individuals. We also expect that higher levels of physical activity would be able to attenuate these effects.

## 2. Materials and Methods

### 2.1. Study Design and Participants

This is a longitudinal study developed in the city of Presidente Prudente, located in the southeastern region of Brazil, with a Human Development Index of 0.806, where 1 is the best and 0 is the worst. The study was conducted according to the guidelines of the Declaration of Helsinki and all procedures used in the study were approved by the Ethics and Research Committee of São Paulo State University (Protocol 019093/2016). All participants signed an informed consent form accepting to voluntarily participate in the study, being duly informed of the research procedures and objectives.

The study sample was composed of female breast cancer survivors. The inclusion criteria were: (1) not having neurodegenerative diseases such as Parkinson’s and Alzheimer’s disease; (2) not presenting other dementias. The exclusion criterion was: (1) not answering all the items in the questionnaire. Participants were recruited from institutions that support breast cancer and by the indication of mastologist doctors from Presidente Prudente. The required sample size was calculated by the formula of Snedecor and Cochran [30]: n = 2 + C(s/d)^2^, where “C” was a constant of 7.85 derived from an alpha error of 0.05 and statistical power 1-β of 0.8, “d” was the mean difference between the averages of the eight QoL domains at baseline and follow-up (3.36), and “s” was the standard deviation of mean difference (9.81), which resulted in a minimum required sample of 69 participants. At the end, a total of 70 women participated in both phases of the study (i.e., baseline and 12-month follow-up).

### 2.2. Data Collection

At first, personal data (name, age, and marital status) and clinical information about the presence of comorbidities, time of diagnosis, whether they had surgery for breast cancer, and what type of surgical procedure, as well as adjuvant treatments, were collected. Body mass and height were assessed by a digital scale with a coupled stadiometer (Welmy^®^, W110H, Brazil). These measurements were used to calculate body mass index (BMI), through body mass (in kilograms) divided by height (in meters) squared.

Thereafter, a face-to-face interview was performed by previously trained researchers. The interview was composed of self-reported instruments to assess depression symptoms, the QoL, habitual physical activity, and socioeconomic status. The participants were assessed at baseline and after 12 months. In both periods, the same assessments were performed. Data collection for all periods of the study ranged from 2016 to 2018.

### 2.3. Depression Symptoms

The Hospital Anxiety and Depression Scale (HADS) was used to assess the presence of depression symptoms. The HADS questionnaire contains 14 multiple-choice questions, which separately assess the symptoms of anxiety (seven questions) and depression (seven questions). Each item scored from 0 to 3, with a higher score representing a greater occurrence of anxiety and depression symptoms. The range for each outcome (i.e., anxiety and depression) is 0 to 21 [32,33,34]. In this study, only the questions related to depressive symptoms were considered, with a score ≥9 corresponding to the presence of depression symptoms [32,33,34]. This cutoff was adopted to classify the sample into “Breast cancer survivors with depressive symptoms (depressive)” and “Breast cancer survivors without depressive symptoms (non-depressive)”. Participants who were classified as breast cancer survivors with depressive symptoms in both evaluations (baseline and 12 months of follow-up) were considered “persistent depressive symptoms”.

### 2.4. Quality of Life (HRQoL)

The Short Form Health Survey (SF-36) questionnaire was used to assess the QoL of the sample. SF-36 was previously translated to Portuguese and validated for the Brazilian population [35,36]. This instrument is composed of 36 items that assess QoL in eight different domains: functional capacity, physical limitations, body pain, general health status, vitality, social aspects, emotional aspects, and mental health. Each domain has a score ranging from 0 to 100, where 0 represents the worst and 100 points represents the best QoL [35,36].

### 2.5. Physical Activity Level

The physical activity level was assessed by Baecke’s questionnaire [37]. This instrument assesses physical activity level (habitual practice) considering the last 12 months in three different domains (i.e., occupational activities, sports practice at leisure, and leisure time and commuting activities) through 16 questions. The occupational activities domain is assessed by eight questions about the physical intensity of the work routine, the frequency of carrying out activities in the work environment (e.g., carrying loads, the need to walk, staying sitting and standing), and if there is fatigue in performing these tasks. The sports practice at leisure domain is evaluated by four questions about the intensity, frequency, and number of hours in sportive and training activities practiced per week, and for how long the activity is practiced. The leisure time and commuting activities domain is determined by four questions about the frequency of walking and cycling for leisure and commuting, as well as the frequency of watching television in leisure time. The Baecke questionnaire provides a dimensionless score for each assessed domain, which ranges from 1 to 5. The overall physical activity score was calculated by the sum of the three domain scores. It should be noted that the Baecke questionnaire is considered to be a valid measure of physical activity level as observed by its good performance when evaluated by gold-standard assessment methods [38].

### 2.6. Socioeconomic Level

The Brazilian Criteria for Economic Classification [39] were used to assess the socioeconomic status of the sample. This instrument considers the educational level and the presence and quantity of specific rooms and consumer goods at home. The questionnaire provides a specific score that classified economic classes from highest to lowest (A1, A2, B1, B2, C1, C2, and D), with “high class” being A1 and A2, “medium class” being B1, B2, and C1, and “low class” being C2 and D [39].

### 2.7. Statistical Analysis

The characteristics of the sample were presented in frequencies, mean values, and standard deviation. Mean differences between QoL scores at baseline and follow-up periods were analyzed by paired t-test. The comparison of domain scores of QoL between persistent depressive and non-depressive groups (not considering the period of assessment) was performed by ANCOVA considering two models. For that, the mean of the value in each domain of QoL at baseline and follow-up was included in the analysis. The first model (Model 1) was adjusted for age, type of surgery, marital status, and socioeconomic status. The second model (Model 2) was composed of Model 1 and physical activity was inserted into this model in order to verify its influence on mean differences. The level of significance was set at *p* < 0.05 and the confidence interval at 95%. All statistical analyses were performed at SPSS Statistical Package, version 15.0.

## 3. Results

Table 1 shows the characteristics of the sample. Most of our participants were married and were classified as having medium socioeconomic status. Regarding the presence of depressive symptoms, 17.1% of the participants from our study (n = 12) were classified as having the persistent presence of depressive symptoms. The most frequent types of surgery and adjuvant treatment were mastectomy and chemotherapy, respectively. Approximately 35% of breast cancer survivors underwent all three types of adjuvant treatment (i.e., chemotherapy, radiotherapy, and hormonal therapy).

The domain scores of QoL at baseline and follow-up are presented in Table 2. Statistical analysis indicated that breast cancer survivors without depressive symptoms improved the scores of QoL in the physical limitations and general health status domains after 12 months (Table 2). No statistical difference was observed between baseline and follow-up (i.e., 12 months) for the QoL domains when considering the breast cancer survivors with persistent depressive symptoms (Table 2).

Table 3 shows the comparison of QoL domains between depressive and non-depressive breast cancer survivors. Regarding Model 1, the statistical analysis demonstrated that breast cancer survivors with persistent depressive symptoms (i.e., participants classified as depressed at baseline and at a 12-month follow-up) present lower QoL values in all domains of the QoL when compared with participants without persistent depressive symptoms (i.e., non-depressive). However, in Model 2 (when including the physical activity level as an adjustment in addition to the confounding factors of Model 1), no significant difference was observed between groups for the functional capacity domain of QoL. The difference between breast cancer survivors with depressive symptoms and participants without depressive symptoms in the other domains of QoL remains significant (Table 3). These results may reflect the possible influence of physical activity level on the functional capacity domain of QoL.

## 4. Discussion

The present study aimed to compare the QoL domains of breast cancer survivors according to persistent depressive symptoms (over 12 months) and analyze the influence of physical activity in the QoL domains in this population. Our results indicated that breast cancer survivors without depressive symptoms improved the QoL (i.e., increased scores) regarding the domains of physical limitations and general health status after 12 months. On the other hand, breast cancer survivors with depressive symptoms did not show significant differences over time. As expected, breast cancer survivors with depressive symptoms showed the worst scores of QoL in all domains when compared with non-depressives, even after adjustment for potential confounding factors (i.e., age, type of surgery, marital status, and socioeconomic status). In addition, after adjusting for physical activity practice, the difference in the scores of the functional capacity domain lost its significance. This result suggests a possible positive effect of physical activity in this QoL domain among breast cancer survivors with depressive symptoms.

Breast cancer survivors with depressive symptoms showed the worst scores in all domains of QoL when compared with women without depressive symptoms. Depression is associated with the individual’s inability to adapt to the stressors of the environment in which they live. Thus, this disorder is characterized by a loss of interest in daily activities, fatigue, pessimism, intense sadness, and guilt [40]. Depression is one of the most prevalent psychological conditions in individuals affected by breast cancer [18]. Despite the well-documented higher prevalence of depression in breast cancer patients, some factors may contribute to the incidence of depression in this population with an impact directly on the QoL. The age (i.e., younger patients), socioeconomic level (i.e., lower income), and level of disease (i.e., severe disease) increase the depression level in breast cancer patients [41]. The time of diagnosis, the type of adjuvant treatment (i.e., chemotherapy), and the number of chemotherapies were related to the presence of depressive symptoms in breast cancer patients [42]. It should be highlighted that depressive symptoms can occur during treatment and also persist after the cure of the disease [18]. In breast cancer survivors, negative thoughts about the future are common and the intensity of such expectations can define the degree of pessimism, which is related to the variability of psychological suffering in this population [18]. Thus, higher degrees of pessimism were associated with a worse QoL in breast cancer survivors [43]. Although the consequences of the disease imply psychological distress, the presence of a previous emotional disorder or concomitant with a physical illness can be considered an aggravating factor for health. Goldney et al. [44] demonstrated that the QoL of individuals with depression was lower than in individuals with the same disease but without depressive symptoms. This result was also evidenced in breast cancer patients [42], mainly in those individuals with several sessions of chemotherapy and more severe disorders [42]. Thus, due to the possibility of depression symptoms persisting after the cure of the disease, which would directly impact the QoL in this population, depression should be considered an important condition to be prevented and treated, especially in breast cancer survivors.

Breast cancer survivors without depressive symptoms improved their QoL regarding the physical limitations and general health domains after 12 months, while participants with depressive symptoms did not show significant differences over time. Breast cancer survivors have had several psychological challenges since the diagnosis of the disease, such as the fear of imminent death, isolation [40], and doubts about the cure [15]. In addition, the physical consequences of treatments to combat breast cancer, such as breast amputation, lymphedema, fatigue, skin changes, pain, and loss of function of the limb homolateral to the surgery, compromise the body image and self-esteem of this population [15,45]. The sum of these factors can contribute to the emergence of mental health issues such as depression [15]. In addition, cancer symptoms associated with depression may be more intense when there is persistent depression, which has a negative impact on physical, emotional, and social health [21]. Moreover, as mentioned previously, depressive symptoms associated with some characteristics of the disease (e.g., number of chemotherapy sessions and disease severity) exacerbate the impairment in the QoL of this population [42]. Such a circumstance may justify the results of this study, since breast cancer survivors who remained depressed over the course of a year presented worse QoL when compared to those without depression.

Although the most of QoL domains remain impaired in breast cancer survivors with depressive symptoms, the “functional capacity” domain seems to be influenced by the level of physical activity in this population. Evidence suggests that changes in lifestyle such as physical activity practice can promote physical [8] and mental [46] benefits, especially in the QoL [8] and depressive symptoms [46] of breast cancer survivors [47]. Likewise, regular practice of physical activity seems to minimize the deleterious effects of breast cancer treatment [46]. Our previous study indicated that the improvement of depression was evidenced mainly in breast cancer survivors with a higher level of physical activity in the leisure/time commuting domain [48]. Breast cancer survivors who participated in a regular program of strength training for six months demonstrated lower depression symptoms and physical fatigue when compared to participants who did not practice strength training [49]. In addition, a recent meta-analysis indicated that mind-body exercise (i.e., activities that combine body movement with mental concentration) may benefit physical fitness, sleep quality, depression, and anxiety [50]. Physical activity programs adapted for the participant’s condition are safe, contribute to the maintenance of a physically active lifestyle, and improve the QoL in breast cancer survivors [47]. The regular practice of physical activity promotes stimulation in several neuroplastic processes involved in cancer disease. For this reason, physical activity helps to reduce the symptoms of depression [28,51] and can contribute to self-esteem and social support [51], which are important aspects of QoL in breast cancer survivors. In addition, physical exercise is associated with reduced inflammation and resistance to oxidative stress [51], factors associated with breast cancer and its recurrence.

Regardless of the attenuation of the functional capacity domains of QoL and depression symptoms in breast cancer survivors, the positive effects of physical activity were not evidenced in other domains of QoL. A possible explanation is that this population has a lower general level of physical activity, since the physical and mental sequelae associated with cancer treatment may result in a reduced engagement in physical activity, which may affect mental health [8,24]. Other factors such as physical and psychological limitations, family responsibilities, lack of transportation, and socioeconomic status have also been reported as important barriers to physical activity [24]. In this sense, evidence about the biological and psychosocial benefits of physical activity may encourage a higher engagement in the practice of physical activity in breast cancer survivors. The lack of knowledge about the benefits of exercise in the sequelae of the breast is one of the barriers to this population’s adherence to physical activity [24]. Thus, we believe that in addition to the adaptation of physical activity considering the participant characteristics, information about the health and benefits of exercise is a key point for maintaining a physically active lifestyle. This is important since physical activity is associated with a better QoL and reduced levels of mental disorders, such as anxiety and depression, among breast cancer survivors [52].

The main limitation of this study is the subjective assessment of physical activity by Baecke’s questionnaire, which did not provide information in minutes per week or account for the intensity of the activities that were performed, precluding more inferences. However, despite this limitation, the Baecke questionnaire is a reliable and valid measurement of habitual physical activity in adults [38]. In addition, the longitudinal design with 12 months of follow-up of the present study, as well as the control of confounding variables in the statistical models, may reduce the chance of possible biases. It is evident that our results indicated that physical activity attenuates the deleterious effect of depressive symptoms on the QoL in breast cancer survivors. Thus, considering that depression is a common mental health issue in general cancer survivors, and that the treatment is specific for each condition, future studies are needed to investigate the influence of physical activity practice in different types of cancer [53].

## 5. Conclusions

Depressive breast cancer survivors have worse QoL than non-depressive women. However, after adjustment for the practice of physical activity, the difference in the functional capacity domain was attenuated, which suggests that physical activity can improve the QoL of breast cancer survivors with depression. This finding reinforces the need for public policies that promote the physical and mental health of breast cancer survivors, including the increase of physical activity as a habitual practice.

## Figures and Tables

**Table 1:** Characteristics of the sample (n = 70).

Variables	n (%) or Mean ± Standard Deviation
Demographic characteristics	
Age, years	50.8 ± 9.7
Body Mass Index, kg/m^2^	28.8 ± 4.7
Physical Activity Level, score	7.8 ± 1.7
Marital status	
Single	7 (10.0)
Married	45 (64.3)
Divorced	7 (10.0)
Widow	11 (15.7)
Socioeconomic status	
High class	11 (15.7)
Medium class	55 (78.6)
Low class	4 (5.7)
Type of surgery	
Quadrantectomy	30 (42.9)
Mastectomy	40 (57.1)
Adjuvant treatment	
Chemotherapy	52 (74.3)
Radiotherapy	44 (62.9)
Hormonal Therapy	41 (58.6)
None	6 (4.2)

**Table 2:** Domain scores of quality of life at baseline and after a 12-month follow-up according to persistent depressive symptoms in breast cancer survivors (n = 70).

	Non-Depressive (n = 58)			Depressive (n = 12)		
	Baseline	Follow-Up		Baseline	Follow-Up	
	Mean (SE)	Mean (SE)	*p*-Value *	Mean (SE)	Mean (SE)	*p*-Value *
Functional capacity	73.6 (3.0)	72.8 (3.2)	0.763	52.7 (6.1)	55.0 (7.1)	0.797
Physical limitations	63.4 (5.8)	81.1 (4.8)	**0.002**	15.9 (9.7)	25.0 (11.2)	0.519
Body pain	60.5 (3.0)	59.9 (2.8)	0.837	33.8 (5.9)	40.4 (5.1)	0.367
General health status	80.1 (2.4)	84.9 (1.9)	**0.037**	56.6 (7.0)	54.6 (7.7)	0.713
Vitality	65.4 (2.5)	62.7 (2.8)	0.325	38.6 (6.5)	44.4 (8.7)	0.439
Social aspects	86.7 (2.9)	89.6 (2.5)	0.331	44.3 (7.2)	43.2 (9.9)	0.867
Emotional limitations	75.3 (5.3)	78.9 (4.9)	0.484	15.1 (9.4)	30.3 (13.8)	0.211
Mental health	75.4 (2.3)	76.0 (2.5)	0.775	48.7 (8.5)	46.9 (8.5)	0.758

* *p*-value of paired *t*-test. SE = Standard error; Bold value indicates significant difference.

**Table 3:** Multiple comparison models of quality-of-life domain scores after a 12-month follow-up between depressive and non-depressive breast cancer survivors (n = 70).

	Non-Depressive (n = 58)	Persistent-Depressive (n = 12)	Model 1		Model 2	
	Mean (SE)	Mean (SE)	*p*-Value *	Effect Size	*p*-Value *	Effect Size
Functional capacity	73.01 (3.36)	53.81 (8.15)	**0.037**	0.067	0.054	0.060
Physical limitations	81.50 (4.66)	23.06 (11.30)	**0.000**	0.258	**0.000**	0.277
Body pain	60.20 (2.71)	38.86 (6.58)	**0.005**	0.120	**0.003**	0.132
General health status	84.70 (2.15)	55.84 (5.20)	**0.000**	0.286	**0.000**	0.285
Vitality	62.80 (2.98)	43.64 (7.22)	**0.019**	0.084	**0.034**	0.072
Social aspects	89.63 (2.81)	43.27 (6.82)	**0.000**	0.375	**0.000**	0.361
Emotional limitations	78.43 (5.04)	32.53 (12.23)	**0.001**	0.155	**0.002**	0.150
Mental health	75.62 (2.75)	48.99 (6.66)	**0.001**	0.172	**0.001**	0.172

* *p*-value of analysis of covariance (ANCOVA). Model 1 = Adjusted for age, type of surgery, marital status, and socioeconomic status; Model 2 = Model 1+ physical activity; SE = Standard error; Bold values indicate significant difference.

## Data Availability

All data are available from the corresponding author upon reasonable request.
